# ReGSP: a visualized application for homology-based gene searching and plotting using multiple reference sequences

**DOI:** 10.7717/peerj.12707

**Published:** 2021-12-23

**Authors:** Girum Fitihamlak Ejigu, Gangman Yi, Jong Im Kim, Jaehee Jung

**Affiliations:** 1 Department of Information and Communication Engineering, Myongji University, Yongin-si, Gyeonggi-do, South Korea; 2 Department of Multimedia Engineering, Dongguk University, Seoul, South Korea; 3 Department of Biology, Chungnam National University, Daejeon, South Korea

**Keywords:** Multiple reference sequences, NGS, Gene, Gene search

## Abstract

The massively parallel nature of next-generation sequencing technologies has contributed to the generation of massive sequence data in the last two decades. Deciphering the meaning of each generated sequence requires multiple analysis tools, at all stages of analysis, from the reads stage all the way up to the whole-genome level. Homology-based approaches based on related reference sequences are usually the preferred option for gene and transcript prediction in newly sequenced genomes, resulting in the popularity of a variety of BLAST and BLAST-based tools. For organelle genomes, a single-reference–based gene finding tool that uses grouping parameters for BLAST results has been implemented in the Genome Search Plotter (GSP). However, this tool does not accept multiple and user-customized reference sequences required for a broad homology search. Here, we present multiple Reference–based Gene Search and Plot (ReGSP), a simple and convenient web tool that accepts multiple reference sequences for homology-based gene search. The tool incorporates cPlot, a novel dot plot tool, for illustrating nucleotide sequence similarity between the query and the reference sequences. ReGSP has an easy-to-use web interface and is freely accessible at https://ds.mju.ac.kr/regsp.

## Introduction

Since their introduction in the mid-2000s, next-generation sequencing technologies (NGS) have greatly increased the amount of available DNA sequence data and their related applications. That is because these technologies incorporate massively parallel sequencing methods that use up to billions of templates simultaneously (McCombie, McPherson & Mardis, 2019). The reduction in sample analysis cost and time is one of the drivers of the observed revolution in DNA sequencing. However, unlocking the information contained in the new DNA sequence data is not trivial and involves several analysis stages, from read assembly at contig level to whole-genome analysis, to annotation and others.

Genome assembly plays an integral role in the interpretation of NGS data, and annotation and other analysis methods are necessary for in-depth genome study. Common annotation pipelines use either *ab initio* gene prediction approaches or homology-based prediction approaches, and sometimes a combination of both, to delineate the gene structure in newly sequenced genomes (Hoff & Stanke, 2015; Ejigu & Jung, 2020). *Ab initio* annotation tools process the newly sequenced genomes to identify specific sequences and signals, such as the start and stop codons, splice sites, polyadenylation sites, etc. (Picardi & Pesole, 2010). Some common *ab initio* tools, such as AUGUSTUS (Stanke et al., 2006), GLIMMER (Delcher et al., 1999), SNAP (Korf, 2004), and Genscan (Burge & Karlin, 1997), predict eukaryotic genes and splicing sites using hidden Markov models (HMMs). *Ab initio* methods are usually the preferred means for the analysis of genomes that lack an identified close relative or a reliable reference genome because they employ statistical analysis to identify gene signals. However, these methods require extensive training and sometimes return errors that could jeopardize subsequent analysis steps, such as gene identification and annotation, especially in draft genomes (Nishimura, Hara & Kuraku, 2019).

On the other hand, homology-based methods operate on the assumption that genes and regulatory sites are conserved to a greater extent than non-functional sequences during evolution. Hence, these methods attempt to predict biological function based on sequence similarity (Wagner, 1989). They rely on a statistically significant similarity between a newly sequenced data and reference sequence, or database, to identify coding DNA segments (Xu & Uberbacher, 1996), transferring the knowledge from one species genome to another.

The most popular method of homology searching involves BLAST (Altschul et al., 1990) from the NCBI public database. After accepting a nucleotide or protein query and/or database sequences *via* a web interface or as a stand-alone application, BLAST attempts to align sequences by heuristically identifying short matches (Johnson et al., 2008). BLAST analysis returns a collection of local alignments called high-scoring segment pairs (HSPs). However, while searching for coding sequences or proteins, BLAST seldom identifies multiple short similar sequences that are scattered throughout the genome because of long and variable introns (Keilwagen et al., 2016). Such similar HSPs are sometimes noise.

Similar homology-based tools, such as exonerate (Slater & Birney, 2005), genBlastG (She et al., 2011), and GeMoMa (Keilwagen, Hartung & Grau, 2019), have been implemented to tackle some of the issues of BLAST. Specifically, exonerate employs bounded sparse dynamic programming (BDSP) to generate gapped alignments from HSPs, with a simple heuristic implementation characterized by better speed and accuracy than those of other dynamic programming algorithms (Slater & Birney, 2005). On the other hand, genBlastG constructs gene structures from candidate genes in HSPs by grouping homologous regions and exploiting sequence signals (She et al., 2011). Similarly, GeMoMa filters genomic regions of HSPs by matching conserved intron positions of specific transcripts and RNA-seq data to predict protein-coding transcripts (Keilwagen, Hartung & Grau, 2019).

The homology-based Genome Search Plotter (GSP) tool was developed to assist the analysis of organellar genomes by identifying genes in published organellar genome data (Jung et al., 2017). GSP uses a reference genome sequence to search for organellar genes based on the grouping of similar fragments, without unnecessary tasks, such as format change. GSP employs BLAST and MUMmer plot (Kurtz et al., 2004) to implement gene search analysis and plotting. However, it only accepts a single reference sequence and does not allow the user to provide own reference sequences. This limits the freedom and flexibility required for homology-based gene searching.

Sequence comparison is an important step for identifying genes and their function. To understand the similarities between sequences, a dot plot visualization method is used for dot matrix analysis of two or more sequences. Following the introduction of the DOTTER (Sonnhammer & Durbin, 1995) program in the late 1990s, well known programs, such as MUMmer (Kurtz et al., 2004) and Gepard (Krumsiek, Arnold & Rattei, 2007), have been employed to understand evolutionary relationships of sequences based on sequence matches, and repeat region and conserved domain analysis.

In the current study, we implemented a modified and simple web tool, the multiple Reference-based Gene Search and Plot (ReGSP) tool. ReGSP can be used during reference-based organellar genome assembly or annotation to identify genes and for similarity analysis of contigs against multiple references, in the protein and nucleotide domains. Similar to GSP, ReGSP uses BLAST to identify HSPs, and relies on two parameters for fragment HSP grouping and contig validation. ReGSP accepts user reference sequences and handles multiple user requests concurrently. In addition to the MUMmer plot, ReGSP is integrated with cPlot, a new dot plot tool (Ji, Jung & Yi, 2021), to enable sequence similarity analysis in the nucleotide domain based on specific *k-mer* size. ReGSP will contribute to organellar genome analysis, with subsequent uses for different analysis levels, such as whole-genome assembly, gene synteny comparison, and annotation.

## Materials and Methods

### Workflow

**Figure 1:**
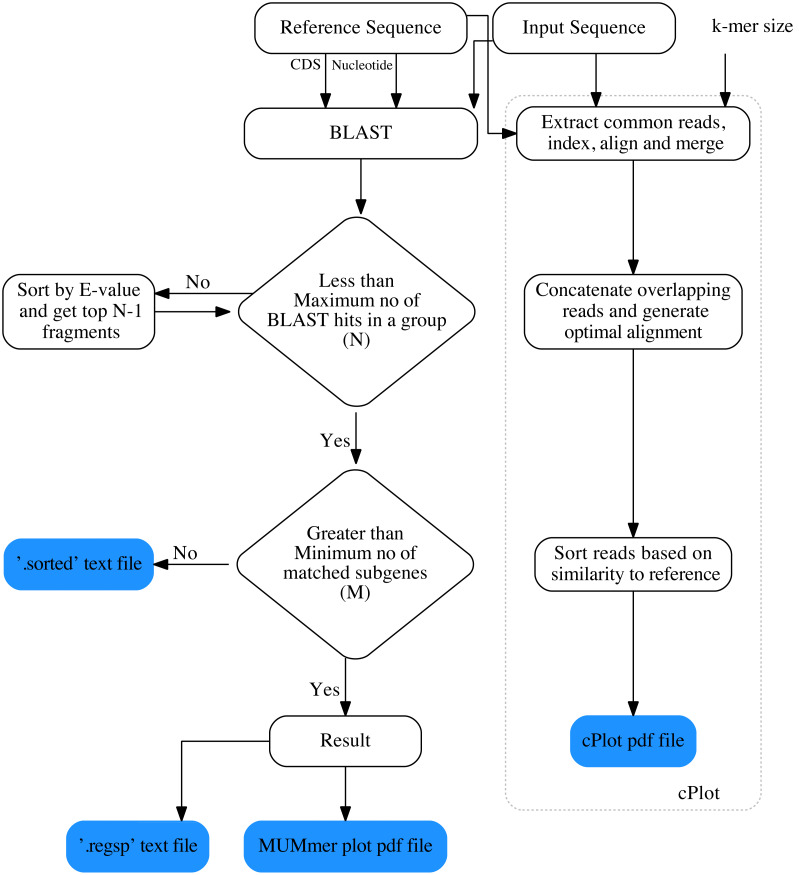
Workfolow of ReGSP.

The overall workflow of ReGSP is shown in Fig. 1. The analysis begins by setting a query and reference sequences by the user. The program then evaluates every query contig against each reference sequence, based on two filtering parameters(see section Gene search parameters), following a BLAST analysis. BLAST is preferentially used for finding HSPs because of its speed and proven ability to find similarity regions (Mount, 2007). ReGSP uses BLASTx tool from the BLAST family, as it performs well for identifying similarities between the sequences of known genes in a reference genome and a provided nucleotide query sequence. Genes that satisfy the two filtering parameters are listed in the final output files, *i.e.,* a .regsp text file and a MUMmer plot PDF file. In parallel, ReGSP performs nucleotide *k-mer* analysis using the cPlot visualization tool. A cPlot PDF file presents a sequence similarity dot plot of all queries plotted against the reference sequences. In addition, ReGSP provides a .sorted text file similar to the .regsp text file, which contains all the results before the second filtering step.

Complete and accurate reference genomes are key to obtaining information on novel genes in a homology search. In some cases, a single reference genome does not provide all the detailed and diverse information required for gene search (Ballouz, Dobin & Gillis, 2019; Yang et al., 2019). Accordingly, ReGSP accepts multiple reference sequences that are phylogenetically close to the query, to validate contigs and identify genes from BLAST fragments obtained after an alignment of each reference and the query. Query sequences can be contigs or scaffolds from newly sequenced or previously published genomes, whenever gene analysis is required.

### Gene search parameters

**Figure 2:**
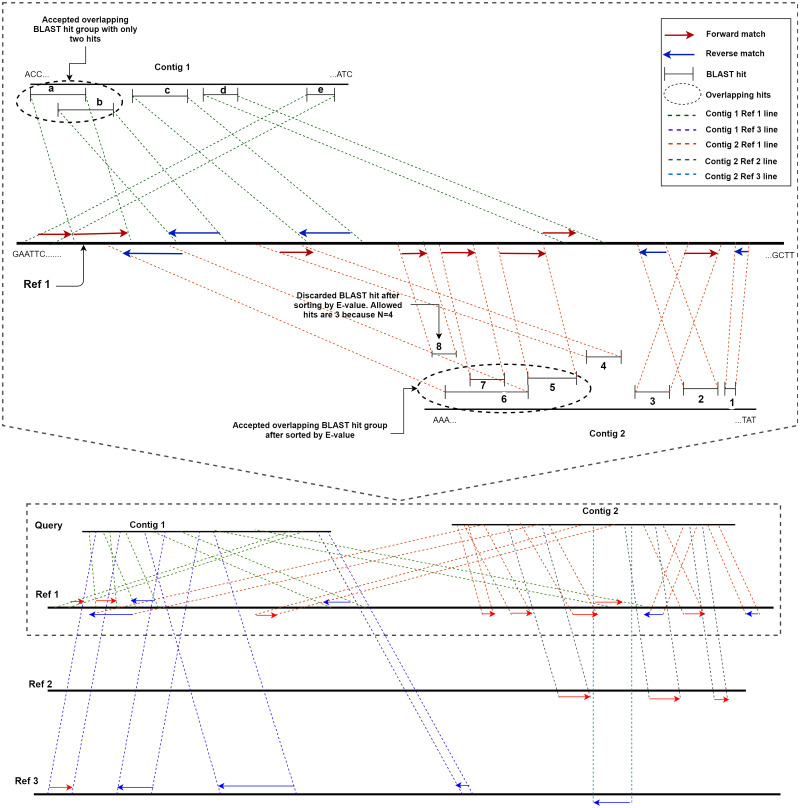
Maximum number of BLAST hits in a group (*N*) and minimum sub gene count per contig (*M*) for values *N,M*= 4. A two-contig query with three reference sequences is shown following BLASTx alignment, with the BLAST hits for Contig 1 shown on top of each reference and Contig 2 hits shown below each reference. Enlarged view of the query sequence comparison with Reference 1 is shown in the top half of the diagram.

ReGSP uses two parameters, the maximum number of matched BLAST hits per group (*N*) and the minimum number of sub-gene counts per contig (*M*), for gene searching. The maximum number of matched BLAST hits per group, *N*, controls the number of allowed overlapping BLAST hits per group, where a group is a non-overlapping section of the contig with a BLAST match against the reference. If a contig search yields overlapping BLAST hits, all matches are considered as one group. Hence, a group can have a maximum of *N* − 1 hits. If the number of hits exceeds this number, the hits will be sorted based on the BLAST score, and the top scoring *N* − 1 hits will be considered to be candidate genes. The second parameter for candidate gene identification is the minimum number of BLAST gene matches per contig, *M*. This parameter dictates the validity of a contig based on the total count of genes in a contig following BLAST analysis and satisfying the first filtering parameter *N*. Note that the genes (matched BLAST hits) in a group are counted individually. If a gene count for a contig does not match the value indicated by *M*, it will not be considered as a candidate gene. To put the two parameters in perspective, let us consider a scenario with two contigs as a query sequence and three reference genomes for which the coding sequences are known. Let us set the first parameter, *i.e.,* the maximum number of matched BLAST hits per group (*N*), as 4 and the minimum number of sub-gene counts (*M*) also as 4. Figure 2 shows a visual representation of the two contigs, in which ReGSP attempts to identify candidate genes.

A detailed view of Reference 1 (top section of Fig. 2) reveals five BLAST hits with Contig 1. The first two matched genes (BLAST match **a** and BLAST match **b**) share an overlapping region; hence, they are considered a group. However, since the maximum number of allowed genes in a group is four, up to three overlapping hits are allowed (*i.e., N* − 1); therefore, both BLAST matches are considered hits. The two overlapping hits and the remaining three hits (**c**, **d**, and **e**) are five sub-gene hits for Contig 1. This qualifies Contig 1 to satisfy the second parameter (minimum sub-gene count), which was set to 4. Similarly, 8 BLAST hits against Reference 1 were detected for Contig 2. Here, BLAST hits **5**, **6**, **7**, and **8** share an overlapping region, and belong to the same group. However, the maximum number of allowed genes per group exceeds the first parameter *N* (a total of 3 allowed, as *N* − 1). Hence, the hits are ordered in a descending manner based on the *E*-value, and the hit (fragment) with the lowest similarity (in this case, BLAST hit **8**) is discarded, with seven BLAST hits remaining. Those are hits **1**, **2**, **3**, and **4**, and the overlapping areas **5**, **6**, and **7**. Contig 2, with seven sub-gene counts, has a higher sub-gene count than that set in the second parameter, which makes it valid.

Consequently, no BLAST hits are identified for Contig 1 against Reference 2, whereas three are identified for Contig 2. For Contig 2, the number of BLAST hits (3) is below that set by *M*; hence, none of the two contigs contain a valid number of genes according to ReGSP. For Reference 3, however, only Contig 1 has a valid set of genes, with four fragments, whereas the single BLAST hit for Contig 2 fails to satisfy the *M* criterion.

Once the genes have been identified for each contig using the two search parameters, ReGSP records the analysis result in a .regsp text file for the user to download. Choosing the appropriate values of the gene search parameters is an adaptive process, and depends on the length or gene content of the references, and the evolutionary distance between each reference and the query.

### Plotting tool

ReGSP uses PROmer to identify regions of conserved protein sequences in a given contig and MUMmer plot to display the results. MUMmer enables rapid alignment of DNA and amino acid sequences, and its graphical module allows assembly viewing, and comparison of alignments of sequences from closely or distantly related species. Hence, in ReGSP, following contig gene validation using the two filtering parameters, a MUMmer plot will reveal conserved regions of contigs against each reference sequence.

Further, ReGSP incorporates a new dot plot tool called cPlot (Ji, Jung & Yi, 2021). This novel tool identifies nucleotide sequence similarity between the provided query sequences and the selected or provided references, to generate a dot plot. Initially, cPlot extracts common reads from the query and reference sequences based on *k-mer* size, and then merges the query sequences that are consecutive when aligned. The aligned reads are *k-mer*–sized and indexed for use at subsequent analysis steps. Next, cPlot identifies an optimal read alignment among the overlapping read candidates (see Fig. 1). To do this, reads that match a provided reference are selected. During this process, the longest base alignment read that is most similar to the reference is selected based on the sorted read index generated at the initial analysis stage. cPlot processes all the reads, starting with the read closest to the region at the beginning of the reference sequence, and collates overlapping reads into a subset. After optimal alignment, the query sequence is rearranged and sorted according to the reference sequence.

cPlot operates in the nucleotide domain, provides additional level of sequence similarity information for ReGSP, and helps to visualize short-read assembly. The original cPlot script was developed to handle a single reference sequence for contig plotting. However, we have modified it to handle multiple references to suit the needs of ReGSP. The final dot plot result of cPlot is provided as a PDF file for downloading.

### Web interface

**Figure 3:**
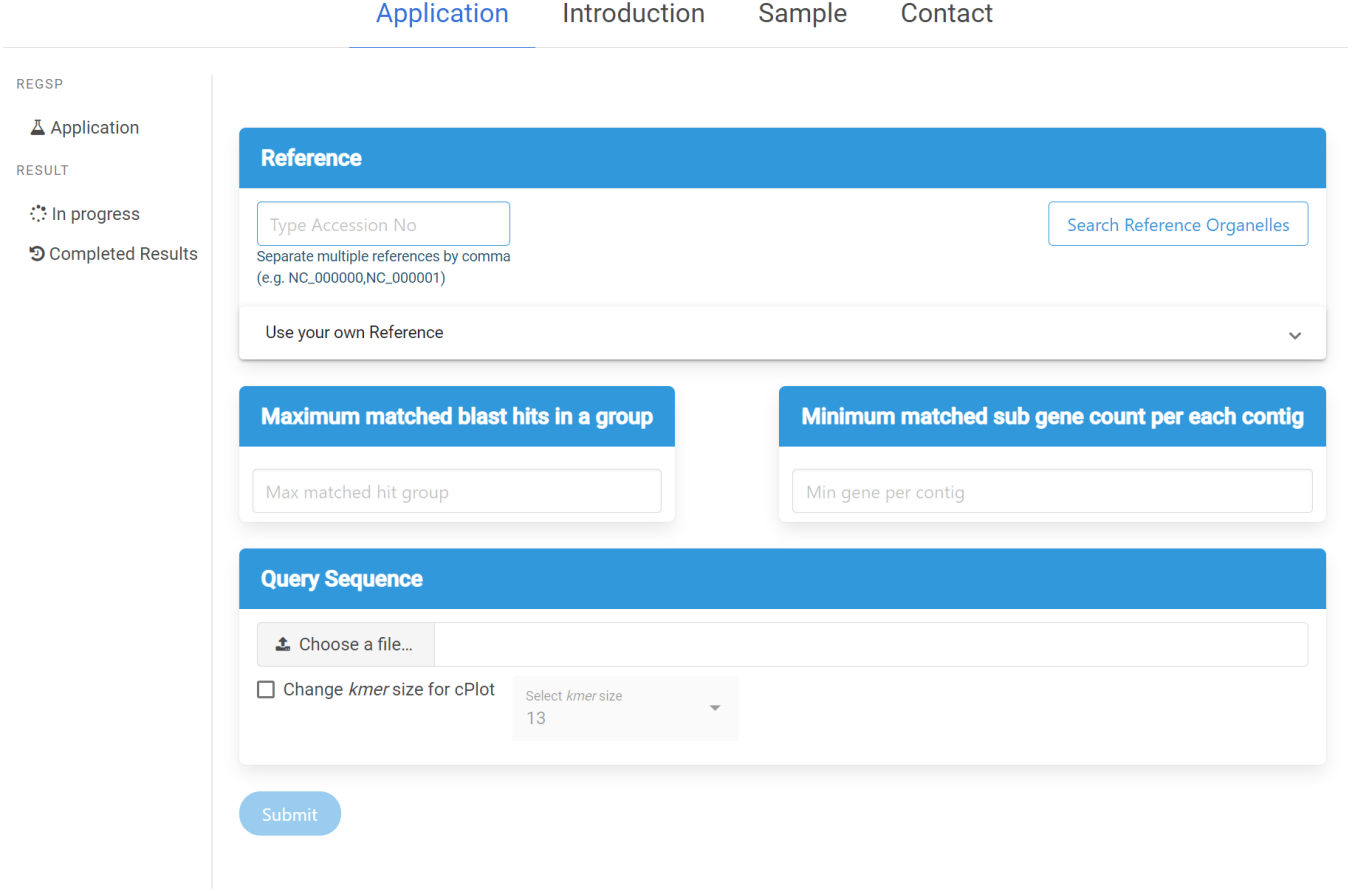
ReGSP web interface.

ReGSP can be accessed at https://ds.mju.ac.kr/regsp. The web interface is freely accessible, and allows anyone who is interested to perform reference-based gene search in a simple and convenient way. The first requirement for using the interface is to provide a reference sequence, and the user has multiple options for reference inputting. The accession number of the reference sequence can be simply typed in a text box. Since ReGSP can handle multiple reference sequences for gene searching, multiple references can be entered separated by a comma. The other option for selecting a reference is using the search tree button, which brings up a list of all organelle genomes from the NCBI Organelle Genome Resources. The organelle search tree contains more than 19,000 organelle genomes, and the user can search and select an organism based on its name. Furthermore, related species from the same genus, family, and kingdom can be selected. On the other hand, if the users want to use their own custom reference sequences, they are required to provide a nucleotide reference sequence or multi-FASTA nucleotide sequences, and an amino acid sequence of the references. The nucleotide and amino acid sequences are uploaded separately. The two input parameters of ReGSP, *N* and *M*, must be set using natural numbers in the two side-by-side text boxes on the interface. The web interface additionally offers an option to select an appropriate *k-mer* size for use in cPlot visualization. A default *k-mer* value of 13 will be used unless the user provides a specific number between 10 and 30. Figure 3 shows the ReGSP interface. Current tasks in progress, completed analysis results, and result history can be accessed on the side menu of the application page. The ReGSP web interface was built using an angular web framework (TypeScript programming language), with a Flask API to communicate with its Python back-end.

### Data set

To evaluate the ReGSP performance, chloroplast genomes of six algal species were analyzed, with four species from the Chromista group and two species from the Rhodophytes. The genomes were retrieved from the public NCBI Nucleotide database. The complete genome of *Heterosigma akashiwo* from the Raphidophyceae clade was used as a query sequence to showcase the analysis. Of note, ReGSP is suitable for analyzing contigs and scaffolds against multiple references. For reference purposes, sequences of two species from the Bacillariophyceae (diatom) class were used: the pennate diatom *Haslea silbo* and *Seminavis robusta*. Another reference was *Nannochloropsis limnetica* from Eustigmatophyceae class. The remaining reference sequences represent two classes of red algae (Rhodophyta) which are *Bangiopsis subsimplex* and *Cyanidium caldarium*.

The dataset can be found in the ReGSP sample page (https://ds.mju.ac.kr/regsp/#/sample), and the details of all the sequences are shown in Table 1. The amino acid sequences for each reference that are used for BLASTx alignments and all the nucleotide sequences including the query were downloaded from NCBI Nucleotide database.

**Table 1:** Data set information.

Sequence	Organism	Accession	Class; Family	Length (bases)
Query	*Heterosigma akashiwo*	NC_010772	Raphidophyceae; Chattonellaceae	159,370
References	*Haslea silbo*	MW645082	Bacillariophceae; Naviculaceae	157,307
	*Seminavis robusta*	MH356727	Bacillariophceae; Naviculaceae	150,905
	*Nannochloropsis limnetica*	NC_022262	Eustigmatophyceae; Monodopsidaceae	117,806
	*Bangiopsis subsimplex*	NC_031173	Stylonematophyceae; Stylonemataceae	204,784
	*Cyanidium caldarium*	NC_001840	Cyanidiophyceae; Cyanidiaceae	164,921

**Figure 4:**
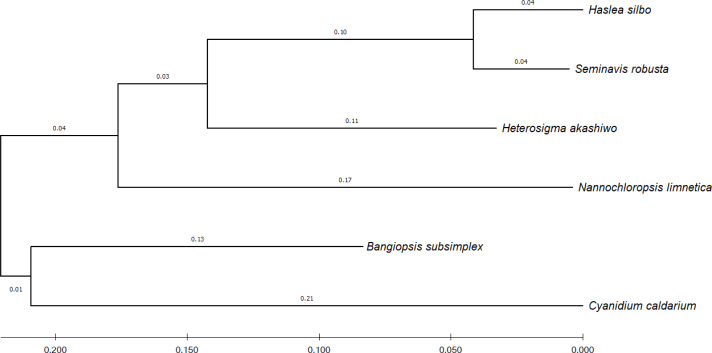
Evolutionary relationship of concatenated sequences of 12 common genes from six algae species. The final aligned dataset of the concatenated genes comprised 16,299 nucleotide positions.

To identify the evolutionary relationship between the query and the reference sequences, 12 common genes harbored by the analyzed algae were selected, namely: *psaA, psaB, psbA, psbB, psbC, atpA, atpB, atpI, petA, petB, rbcL,* and *rpl2*. According to UniProtKB (Consortium, 2021), the *psa* and *psb* gene families encode protein subunits of photosystem I (PSI) and photosystem II (PSII), respectively, which drive oxygenic photosynthesis, whereas *petA* and *petB* encode components of the cytochrome b6-f complex, which mediates electron transfer between PSI and PSII. The genes *atpA, atpB,* and *atpI* are involved in the production of ATP, while the *rbcL* gene catalyzes carbon dioxide fixation and *rpl2* is ribosomal protein large subunit involved in translation. For each species, the sequences of the 12 genes were combined and subsequently aligned using the MUSCLE program in MEGA X (Kumar et al., 2018). Figure 4 shows the phylogenetic tree constructed based on these genes, with their evolutionary history inferred using maximum-likelihood method and Tamura–Nei model (Tamura & Nei, 1993).

## Results

ReGSP generates three main output files and an additional .sorted text file after analysis. We used the chloroplast genome of *Heterosigma akashiwo* (NC_010772) as the query sequence. We then compared *Heterosigma akashiwo* sequence with those of five red-algal derived species: three secondary or tertiary evolved red-algal derived species, Stramenopiles (*i.e., Haslea silbo, S. robusta,* and *N. limnetica*) and two from red algae (*B. subsimplex* and *C. caldarium*). The details of the sequences are presented in Table 1. We uploaded both, a multi-FASTA nucleotide sequence and CDSs of all references to the ReGSP web interface. When providing multiple reference CDSs, ReGSP needs a minimum of three asterisk (***) separator between each references. We set *N* and *M* parameters as 1 and 50, respectively, which did not allow overlapping results, with a minimum of 50 BLAST hits per contig. Finally, we tested four *k-mer* sizes independently, including the default *k-mer* size of 13, for the review of the cPlot result before submitting the task.

### Text file output

ReGSP presents homology-based gene search results for the submitted query against the provided references in two text files (.regsp and .sorted). In the current study, all counts of gene matches for the *Heterosigma akashiwo* query against the references extracted from the .sorted text file are shown in Table 2. Customized and sorted BLASTx output is shown in the .sorted text file, before proceeding with the second parameter analysis, *i.e.,* the minimum gene count per contig (*M*). The .regsp text file is similar to the .sorted text file, except that it includes the output after *M* parameter analysis. As shown in Table 2, the minimum number of genes per contig was greater than 50 for all references. Hence, in this case, the .regsp and .sorted text files showed identical results. However, for cases where the number of hits for contigs and/or scaffolds is smaller than *M*, the two text files show different gene counts. Further, reference sequences that are phylogenetically close to the query sequence have a higher number of BLAST hits than those for distant relatives with similar base length.

**Table 2:** Matched gene counts *M* of *Heterosigma akashiwo* against five algae references.

Query details	Reference details and hit count				
*Heterosigma akashiwo*	*H. silbo*	*S. robusta*	*N. limnetica*	*B. subsimplex*	*C. caldarium*
156 CDSs	161	160	136	153	152

**Figure 5:**
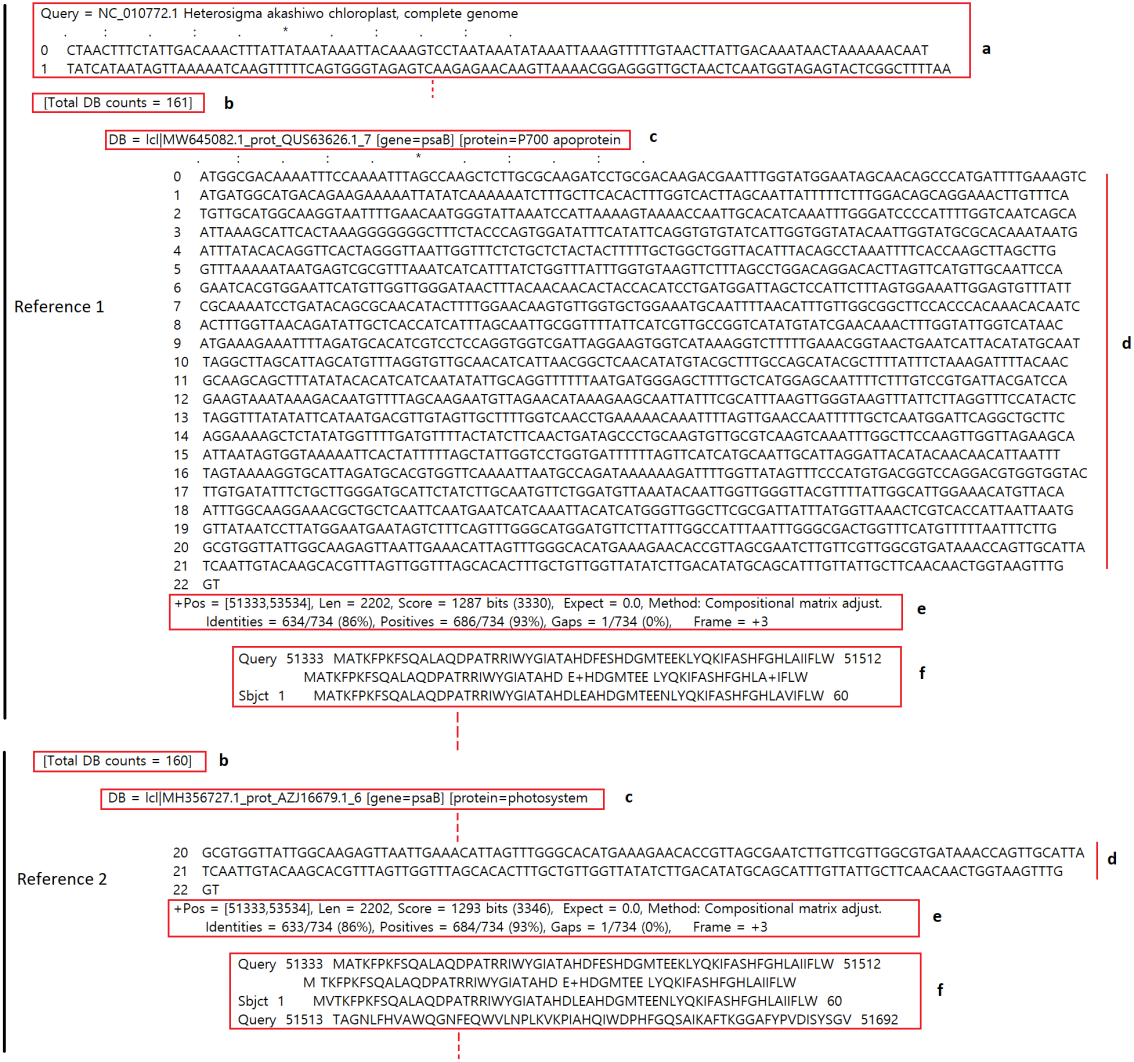
Partial view of .regsp text file data. Details of *Heterosigma akashiwo* gene alignment against the first two references are highlighted: **a**, query sequence; **b**, total count of contig genes against a reference; **c**, gene information; **d**, nucleotide sequence of the query matching the gene; **e**, detailed BLASTX information, such as sequence position in the query, alignment length, bit score, and translation frame; and **f**, alignment of each gene with translated query.

In addition to the number of gene hits, the two text files contain detailed gene information, such as sequence hit region, alignment length, bit score, translation frame, and the resulting protein alignment. These details provide valuable information for the analysis of the query sequence against the reference(s), and are sorted based on similarity to each reference. Figure 5 shows a small section of the .regsp text output. The various pieces of information were retrieved for all references, and gene search results were sorted based on similarity score.

### Plot output

**Figure 6:**
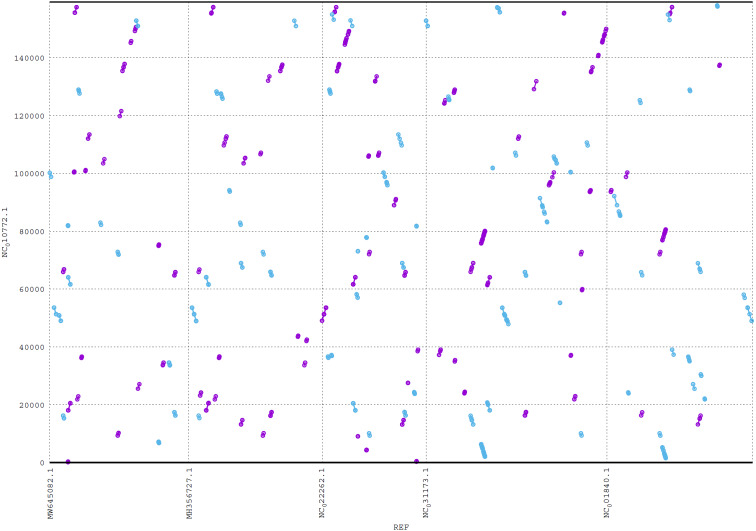
MUMmer plot output for *Heterosigma akashiwo* gene search against five algae reference sequences. Purple color indicates forward matches and cyan indicates reverse matches in PROmer alignment.

ReGSP generates two plot files. The first plot file is generated using the PROmer alignment program of MUMmer. For the analysis, all query contigs are aligned against each provided reference sequence in all (six) amino acid frames to reveal conserved regions in the sequences. The high sensitivity of PROmer leads to an improved alignment of sequences that are positioned close in the phylogenetic tree. Using the *Heterosigma akashiwo* query example, the analysis detected multiple conserved genes in all reference sequences, with a slight preference for species more closely related to the query, as shown in Fig. 6 and the phylogenetic tree in Fig. 4. Figure 6 shows the alignment of *Heterosigma akashiwo* query genome sequence, with every reference in a separate grid.

**Figure 7:**
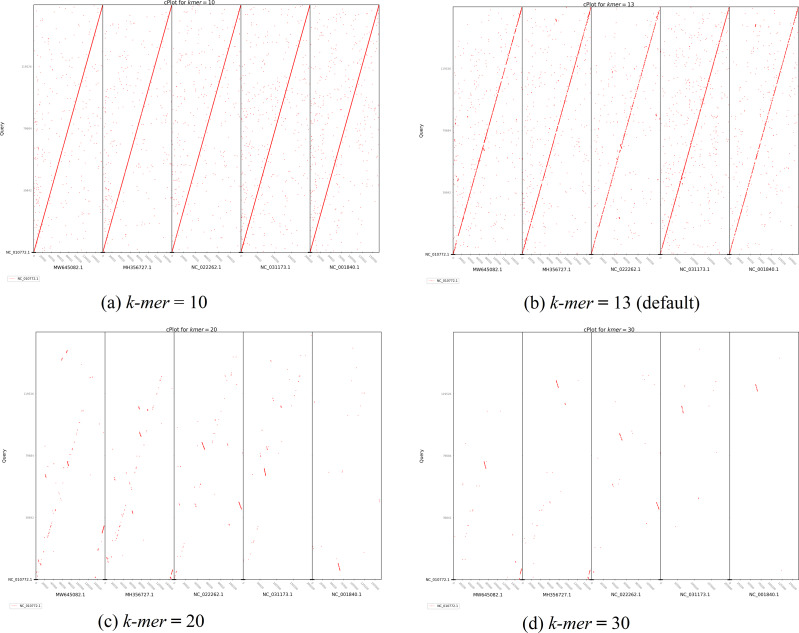
cPlot output for *Heterosigma akashiwo* gene search with five red-algal derived plastid containing algae reference sequences. Each panel (A–D) shows the results at different *k-mer* value used for nucleotide sequence similarity determination.

The second plot output of ReGSP is a nucleotide domain dot plot generated by cPlot. Figure 7 presents different *k-mer*–size plots of the *Heterosigma akashiwo* chloroplast genome with the provided reference sequences: a *k-mer* value of 10 (Fig. 7A) and a *k-mer* value of 13, the default *k-mer* value for ReGSP (Fig. 7B). Typically, multiple instances of sequence similarity are identified at small *k-mer* values for species that are evolutionarily close to the query, as well as those that are distant from the query. Small *k-mer* values are helpful when most of the references are distantly related to the query. However, as the *k-mer* value increases, only highly similar sequence alignments are detected, and the dot plot fades out for less similar sequences. Figures 7C and 7D illustrate this property, at *k-mer* values of 20 and 30, respectively. Therefore, high *k-mer* values reveal long segments of sequence similarity, with dot plots available for sequences that are closely related. On the other hand, low *k-mer* values, which require a relatively longer ReGSP execution time, will result in multiple short matched segments. For the *Heterosigma akashiwo* example, the query shares high sequence similarity with *Haslea silbo* and *S. robusta* sequences, which belong to the same clade it occupies. Further, the *Heterosigma akashiwo* chloroplast sequence shared the lowest similarity with algae from the Plantae kingdom. The ReGSP analysis findings concur with the phylogenetic relationship of the species, as shown in Fig. 4.

Finally, based on testing of different lengths of query and reference sequences, for most optimized visual plot results, we recommend providing no more than 30 multi-FASTA files as aquery and less than 10 reference sequences.

## Discussion

Reference-based gene search is a quick and cost-effective approach for gene prediction and annotation in newly sequenced genomes. GSP (Jung et al., 2017) is a gene prediction and annotation tool that generates contig gene information using the parameters *N* and *M*. However, the main constraints of GSP are the inability to accept multiple references and to allow the user to provide own custom reference sequences, thus limiting the flexibility of analysis. ReGSP, on the other hand, was designed to allow the use of custom and multiple references. Further, unlike GSP, which uses only protein domain MUMer plot, ReGSP implements a novel dot plot visualization tool that compares the provided query contigs against the reference sequences (in the nucleotide domain), according to a user-selected *k-mer* value, for additional sequence similarity analysis. In addition, while GSP lacks the feature to accept multiple requests, ReGSP accepts concurrent tasks because of a Python back-end that supports multi-threading. The user can view and download the output files once the specific tasks are completed from the task history list. Table 3 shows a simple feature comparison of GSP and ReGSP.

**Table 3:** Feature comparison of GSP and ReGSP.

	Homology analysis	Multiple/Custom reference	Nucleotide-domain plot (cPlot)	Multiple task	History
GSP	Yes	No	No	No	No
ReGSP	Yes	Yes	Yes	Yes	Yes

When it comes to performance, direct performance comparison of ReGSP and GSP is not informative since ReGSP contains the integrated new cPlot module. However, we implemented ReGSP using the method suggested in Jung & Yi (2019). ReGSP employs the whole-targeted read method recommended by Jung & Yi (2019), with a BLAST execution time shorter than that of GSP. Hence, ReGSP already performs better than GSP when only a single reference genome is considered.

ReGSP’s web tool offers researchers to perform homology-based gene analysis without concerning themselves about program and package dependencies needed to use the tool. However, some scientists may not want to use a web tool for their unpublished data. To address such a concern, ReGSP has an alternative binary package that can be executed from a Linux local machine. When using this executable, all inputs required for a task execution have to be provided from a Linux terminal. The executable binary package of ReGSP is available at ReGSP webpage.

## Conclusions

Multiple reference-based gene search and plot program (ReGSP) is a reimplementation of GSP that simplifies the task of homology-based gene search and sequence similarity analysis of NGS contigs. ReGSP identifies genes in contigs using two parameters whose value is set by the user: the maximum number of BLAST hits per group and the minimum number of BLAST genes per contig. These parameters are employed following a BLAST search against a reference sequence to identify valid coding DNA segments. ReGSP is not restricted to a single reference sequence, however. In fact, the user can select multiple references and supply multiple custom references. Further, cPlot, a novel plotting tool, was integrated in ReGSP and calculates nucleotide sequence similarity for a selected *k-mer* value. Finally, the simple web interface of ReGSP enables easy and convenient organelle contig analyses.

In summary, the ReGSP text and plot output files present homology-based gene search results and query sequence similarity with specific references. However, in the future, ReGSP could be developed further, providing an interactive graphical output to allow the user to zoom in, focus, and analyze contigs in particular reference areas, and even make annotations. Further, it can be modified to accommodate nuclear genomes that are much larger in size. Nonetheless, ReGSP is able to provide homology-based gene search and plot for organellar genomes from provided references conveniently. ReGSP is widely available at https://ds.mju.ac.kr/regsp.

## Additional Information and Declarations

### Data Availability

The following information was supplied regarding data availability: ReGSP is available at: https://ds.mju.ac.kr/regsp. The datasets are available at NCBI: NC_010772, MW645082, MH356727, NC_022262, NC_031173, NC_001840.
